# Pathogenic p.Cys194Metfs*17 variant argues against *TMPRSS3/GJB2* digenic inheritance of hearing loss

**DOI:** 10.1007/s00405-015-3782-7

**Published:** 2015-09-25

**Authors:** Urszula Lechowicz, Agnieszka Pollak, Dominka Oziębło, Monika Ołdak

**Affiliations:** Department of Genetics, Institute of Physiology and Pathology of Hearing, M. Mochnackiego 10, 02-042 Warsaw, Poland

Dear Editor,

We read with great interest the article “*TMPRSS3* mutations in autosomal recessive nonsyndromic hearing loss” by Battelino et al. [1]. Performing targeted next-generation sequencing the authors have identified *TMPRSS3* mutations as a frequent cause of autosomal recessive non-syndromic hearing loss (ARNSHL) in Slovenian patients and demonstrated that *TMPRSS3* gene analysis should be performed in the first tier of ARNSHL genetic investigations [1]. While this is an interesting and clinically relevant finding, our attention was drawn by the interpretation of the genetic results obtained in one individual of the index family. This patient with profound congenital deafness was identified to be compound heterozygous for c.208delC and c.579dupA mutations in the *TMPRSS3* gene (DFNB8/10) and heterozygous for the c.35delG mutation in *GJB2* (DFNB1), which is the most common cause of ARNSHL in Caucasian population [2]. Battelino et al. have classified *TMPRSS3* c.579dupA as a non-pathogenic variant located in the noncoding exon and based on the presence of the two other mutations (c.208delC in *TMPRSS3* and c.35delG in *GJB2)* implied a possible *TMPRSS3/GJB2* digenic inheritance of ARNSHL in this family.

It is not clear for us why the authors did not accept *TMPRSS3* c.579dupA as a causative variant. We could not find the *TMPRSS3* reference sequence in the paper by Battelino et al. but based on the nomenclature used (c.579dupA and p.Cys194Metfs*17, rs397517376), we assume that they refer to the NM_024022.2 and NP_076927.1 sequences at the mRNA and protein levels, respectively. According to these sequences c.579dupA localizes to the *TMPRSS3* coding region. This frame-shift variant introduces a premature stop codon, which most probably severely truncates the protein and has a deleterious effect on its function. *TMPRSS3* c.579dupA is reported in the Single Nucleotide Polymorphism Database (dbSNP) and ClinVar as pathogenic (http://www.ncbi.nlm.nih.gov/clinvar/, http://www.ncbi.nlm.nih.gov/projects/SNP/, accessed 08/2015) and our data also support its pathogenic potential (U. Lechowicz [3], manuscript in preparation). Furthermore, two other *TMPRSS3* mutations (NM_024022.2:c.582T>A; NP_076927.1:p.C194* and NM_024022.2:c.581G>T; NP_076927.1:p.C194F) were detected in the region corresponding to the same codon position (p.Cys194) in Palestinian and Pakistani families with ARNSHL, respectively [4, 5].

Deafness is an excellent candidate for digenic inheritance with examples for non-syndromic and syndromic hearing loss [6]. Both, *GJB2* and *TMPRSS3* are expressed in the supporting cells and stria vascularis of the inner ear [7] but except for a frequent co-occurrence of both gene names in scientific articles, in silico analysis does not suggest any functional link between these proteins (STRING 10.0, http://string-db.org/). To the best of our knowledge, there is neither in vivo nor in vitro data supporting the hypothesis of *TMPRSS3/GJB2* digenic inheritance of deafness and the results provided by Battelino et al. are, in our opinion, also insufficient to reinforce it. We are strongly convinced that ARNSHL in the reported patient is a consequence of *TMPRSS3* compound heterozygous c.208delC and c.579dupA mutations and in addition to that the patient is also a carrier of c.35delG in *GJB2.* Whether *GJB2* mutations may modify the phenotype of ARNSHL in patients with biallelic *TMPRSS3* mutations remains to be determined.
